# Central effects of galcanezumab in migraine: a pilot study on Steady State Visual Evoked Potentials and occipital hemodynamic response in migraine patients

**DOI:** 10.1186/s10194-022-01421-z

**Published:** 2022-04-29

**Authors:** Marina de Tommaso, Marianna La Rocca, Silvia Giovanna Quitadamo, Katia Ricci, Giusy Tancredi, Livio Clemente, Eleonora Gentile, Elena Ammendola, Marianna Delussi

**Affiliations:** 1 Applied Neurophysiology and Pain Unit, Bari Aldo Moro UniversityPoliclinico General Hospital, Piazza Giulio Cesare 11, 70124 Bari, Italy; 2 Dipartimento Interateneo di Fisica ‘M. Merlin’, Università degli Studi di Bari ‘A. Moro’, Bari, Italy; 3 Laboratory of Neuro Imaging, Stevens Neuroimaging and Informatics Institute, Keck School of Medicine, University of Southern California, Los Angeles, CA USA

**Keywords:** Migraine, CGRP, galcanezumab, steady state visual evoked potentials, fNIRS

## Abstract

**Background:**

The discovery of the prominent action of Calcitonin Gene Related Peptide –CGRP- on trigeminal afferents and meningeal vessels, opened a new era in migraine treatment. However, how the block of nociceptive afferents could act on central mechanisms of migraine is still not clear. In this pilot study we aimed to test the effect of 3 months Galcanezumab (CGA) therapy on occipital visual reactivity in migraine patients, using the Steady State Visual Evoked Potentials-SSVEPs and Functional Near Infrared Spectroscopy –fNIRS.

**Method:**

Thirteen migraine patients underwent clinical and neurophysiological examination in basal condition (T0), 1 h after GCA injection (T1) and after 3 months of GCA treatment (T2). Ten healthy volunteers were also evaluated.

**Results:**

At T2, there was a reduction of headache frequency and disability. At T2, the EEG power significantly diminished as compared to T0 and T1 at occipital sites, and the topographical analysis confirmed a restoration of SSVEPs within normal values. The Oxyhemoglobin levels in occipital cortex, which were basically increased during visual stimulation in migraine patients, reverted to normal values at T2.

**Conclusions:**

The present pilot study indicates that Galcanezumab could act on cortical targets located beyond the pain network, restoring the abnormal occipital reactivity. This effect could indicate the possible disease modifying properties of CGRP related monoclonal antibodies.

## Background

The discovery of the prominent role of the Calcitonin Gene Related Peptide (CGRP) in the trigeminal-vascular system, has opened a new era in migraine treatment [1]. Monoclonal antibodies (mABs) directed against CGRP receptors, are large molecules which inhibit the a-delta meningeal and perivascular fibers (responsible for CGRP receptors) in the periphery, blocking the vascular dilation and the development of sterile inflammation [2]. Their efficacy has been well established in several randomized controlled trials, which recently indicated a long-term effect, up to 5 years treatment [3, 4].

However, how a peripheral block of nociceptive afferents could modify migraine outcome and act on basal central mechanisms of attack onset is still not clear.

Recent studies on the effect of CGRP antagonists on central responses to nociceptive stimulation, found an inhibition of cortical regions devoted to pain processing [5], and involved in migraine attack generation [6]. The inhibition of cortical networks activated by the nociceptive action of CGRP, could also interest cortical areas outside the pain matrix. In fact, the cortical areas involved in the hypersensitivity to multimodal stimuli, the so called “allostatic load”, could be modulated by an inhibition of the circuits causing headache pain [7].

Several lines of evidence indicate that migraine is characterized by an altered visual cortex excitability both during and between attacks [8]. Electroencephalography (EEG) studies have largely demonstrated that brain oscillations under multimodal and particularly visual stimuli are different in migraine patients compared with controls [9, 10]. In particular, visual responses evoked by black and white checkerboard at 5 Hz temporal and 0.5 cpd spatial frequencies (Steady State Visual Evoked Potentials or SSVEPs), were different in interictal migraine with and without aura compared to controls. The 2th harmonic (10 Hz) power was found increased in both migraine groups [11].

Our group used an analogues type of stimulation and found a different pattern of cortical connections in migraine compared to controls [12].

However, there is no evidence of a direct effect of anti CGRP agents or sumatriptan on visual cortex, for a prevalent action outside of the blood-brain barrier [13].

The Functional Near Infrared Spectroscopy (fNIRS) allows the study of brain metabolism study and could be an efficient method to reveal the abnormal cortical activity in migraine. Like functional magnetic resonance imaging (fMRI), fNIRS detects the changes of hemoglobin inside the brain through the measure of differences in optical absorption [14]. The concurrent EEG-fNIRS recording offers a useful, low cost and noninvasive method to detect bioelectrical and hemodynamic changes during brain functions [15]. In fact, fNIRS data showed an abnormal hemodynamic activity of the visual cortex in migraine patients stimulated with a checkerboard pattern reversal [16].

We wonder if the medium-long term modulation of CGRP neurotransmission, could modify the visual cortex activity and predispose migraine mechanisms.

In this aim, we tested the effect of 3 months treatment with Galcanezumab-GCA, a monoclonal antibody directed to CGRP, on the SSVEPs and fNIRS features obtained with 5 Hz and 0,5 cpd checkerboard in a cohort of patients with drug resistant migraine.

This pilot study was carried out within the routine clinical practice, and it did not include a placebo session. A recording session soon after galcanezumab injection, served as control.

## Methods

### Subjects

This was a pilot study based on the neurophysiological effects of 3 months galcanezumab therapy in drug resistant migraine. Migraine patients were selected at the tertiary Headache Center of Applied Neurophysiology and Pain Unit of Bari Policlinico General Hospital from December 2020 to April 2021, during the routine clinical practice. In agreement with Italian rules for drugs reimbursement, only patients with high frequency drug resistant migraine could receive CGRP monoclonal antibodies prescription. Therefore, selection criteria for Galcanezumab treatment were: migraine diagnosis, according to current criteria (migraine with aura, without aura or chronic migraine) [17], 8 days or more with migraine / month in the last 3 months, resistance to at least three preventive drugs, including or not botulinum toxin for chronic migraine. The ongoing preventive treatment was thus suspended for inefficacy at the time of galcanezumab prescription. All the selected chronic migraine patients were assuming symptomatic drugs-triptans and NSAIDs for more than 10 days/month at the time of study inclusion. However, no patient had received the diagnosis of associated Medication Overuse Headache (MOH), the temporary reduction of symptomatic drugs, treatment changes for abuse, or past detoxification treatments that could have modified migraine characteristics. There were no specific exclusion criteria for Galcanezumab prescription, except for not controlled hypertension, ischemic heart failure, recent or previous history of stroke or TIA, thromboembolic events, aortic bypass or other type of vascular surgery. EEG and fNIRS recording were performed during the migraine-free periods, and an interval of 24 h from the last and the next migraine attack, ascertained with telephonic interview, was requested. Longer migraine free intervals wouldn’t be satisfied as inclusion criteria in such severe patients. We took special attention in recording patients during similar migraine-free intervals in basal condition at T0 and after 3 months galcanezumab at T2. Thirteen migraine patients (6 with chronic migraine) were finally included, among the 15 patients initially screened. One patient did not give the consent to the EEG/fNIRS recording, 1 patient was Covid positive at the evaluation after 3 months (T2). Demographic and clinical data of the selected patients are detailed in Table 1. All patients filled headache daily records reporting days with headache and its intensity as detailed in previous studies [18]. Briefly, intensity of headache was evaluated through a numerical rating scale from 0 to 10, frequency of headache was the average number of days with headache in a month, computed in 3 months. We applied the Italian version of MIDAS before and after three months treatment with galcanezumab [19].

**Table 1:** Demographic and clinical data of migraine patients at the basal visit (T0) and after 3 months of galcanezumab (240 mg initial dose, 120 mg in the 2nd and 3rd month) (T2) therapy. MA: episodic migraine without aura; CM Chronic Migraine; AED antiepileptics-topiramate and/or valproate; AD antidepressants-amitriptyline; BB beta blockers-propranolol or atenolol; CA calcium channel blockers-flunarizine; TB Botulin Toxin. Frequency was computed as average over 3 months of the number of headache days per month (DAYS/30), symptomatic drug taking was evaluated as average over 3 month of the number of days per month with acute therapy (SIM/30). Headache intensity was expressed with a Numerical Rating Scale –NRS- from 0 no pain to 10 maximal tolerable pain. At the bottom, results of Student’s t test between T0 (basal condition) and T2 (after 3 months of GCA treatment) are reported

	GENDER	AGE	DURATION	DIAGNOSIS	DRUGS	T0	DAYS/30	SIM/30	MIDAS	NRS	T2	DAYS/30	SIM/30	MIDAS	NRS
AI	F	29	20	MA	AED, AD, BB	2	13	13	79	10		17	16	113	10
BA	F	48	30	MA	AED, AD, BB,TB	1	12	12	26	7		8	8	12	4
DD	F	38	22	MA	AED,AD,CA	1	9	9	68	10		4	4	34	10
DE	F	47	25	CM	AED,AD,CA,TB	2	17	17	150	9		17	10	70	6
DI	F	48	25	MA	AED,AD,CA,BB	1	13	13	26	7		10	10	13	6
DL	F	47	24	MA	AED,AD	1	8	12	32	10		3	3	10	6
FA	F	57	40	CM	AED,AD,CA,TB	1	20	20	75	7		4	4	12	8
LE	F	42	30	CM	AED,AD,CA,BB,TB	1	27	27	30	8		6	6	10	6
MA	F	67	50	CM	AED,AD,CA,BB,TB	2	15	4	50	8		15	3	25	5
MO	F	47	30	CM	AED,AD,TB	1	25	25	56	8		12	10	25	7
MN	F	63	45	MA	AED,AD,CA,BB	1	10	12	5	6		5	0	0	4
PN	F	58	10	MA	AED,BB,AD	1	13	13	77	7		8	8	33	7
SM	M	56	30	CM	AED,BB,AD	1	15	15	108	10		5	5	50	8
*Mean (SD)*							*15.5 (8.7)*	*14.7 (6.2)*	*60.1 (39.2)*	*8.2 (1.4)*		*8.7 (5)*	*6.6 (4.2)*	*31.3 (31)*	*6.6 (2)*
*T test*							*3.3*	*4.3*	*3.59*	*3.68*					
*p*							*0.006*	*0.001*	*0.004*	*0.003*					

We planned to record EEG and fNIRS data for 13 controls, who did not have history of medical and neurological diseases, including migraine. In 2 cases we had technical problems for the presence of incorrigible artifacts in the fNIRS data, 1 subject reported migraine attacks in the weeks following the experiment. Ten age and sex matched healthy volunteers (1 male, mean age 48.8 + 9.9) were finally considered for the EEG and fNIRS analyses. Controls were recorded once.

### Steady state visual evoked responses (SSVEPs)

Stimulation and recording - Black and white checkerboard patterns were presented on a 17-in. monitor subtending 21 × 17° at a viewing distance of 90 cm during EEG recordings. The spatial frequency of 0.5 cycle per degree (cpd) was used. The mean luminance was 14 cd/m2. The stimulus pattern was alternated at 5 Hz (10 reversal/s). The stimulation lasted 60 s. We performed only 2 consecutive sessions of checkerboard pattern stimulation, to avoid a long and stressful procedure and a possible occurrence of acute migraine. EEG data were acquired simultaneously with fNIRS data, using a co-recording cap and a black over-cap to mitigate possible interferences generated by ambient light on the fNIRS acquisition. EEG data were recorded by 62 scalp electrodes, according to the enlarged 10–20 system, referring to the nasion with the ground at Fpz. Impedance was below 5000 Ω. Two electrodes were placed above the right and left eye to record the EOG. The sampling rate was 256 Hz.

### EEG analysis

The SSVEPs were examined blind for the study phases (T0, T1, T2). Preprocessing was performed in MATLAB using the EEGLAB 14_1_1, for the conversion of the original EEG files. Bad channels were identified and removed by a semi-automatic method based on visual detection and channel statistics. Channels presenting distributions further away from the Gaussian distribution were also deleted.

We thus uploaded the EEGs in the letswave matlab tool, removed ocular artifacts recorded on EOG channels [20], and applied a notch filter at 50 hz. After this, we estimated the spectral power using the FFT-Fast Fourier Transform, averaging 12 samples of 10 s for the 2 recording sessions, and applied a baseline correction in the frequency domain.

The power of fundamental frequency (F) at 5 Hz and double frequency (2F) at 10 Hz were considered. We also observed and computed a third frequency at 15 Hz (3F).

We performed a single channels analysis (O1, O2, Oz) and a topographical analysis in the considered frequencies, according to Matlab Letswave software.

### fNIRS data acquisition

**Fig. 1:**
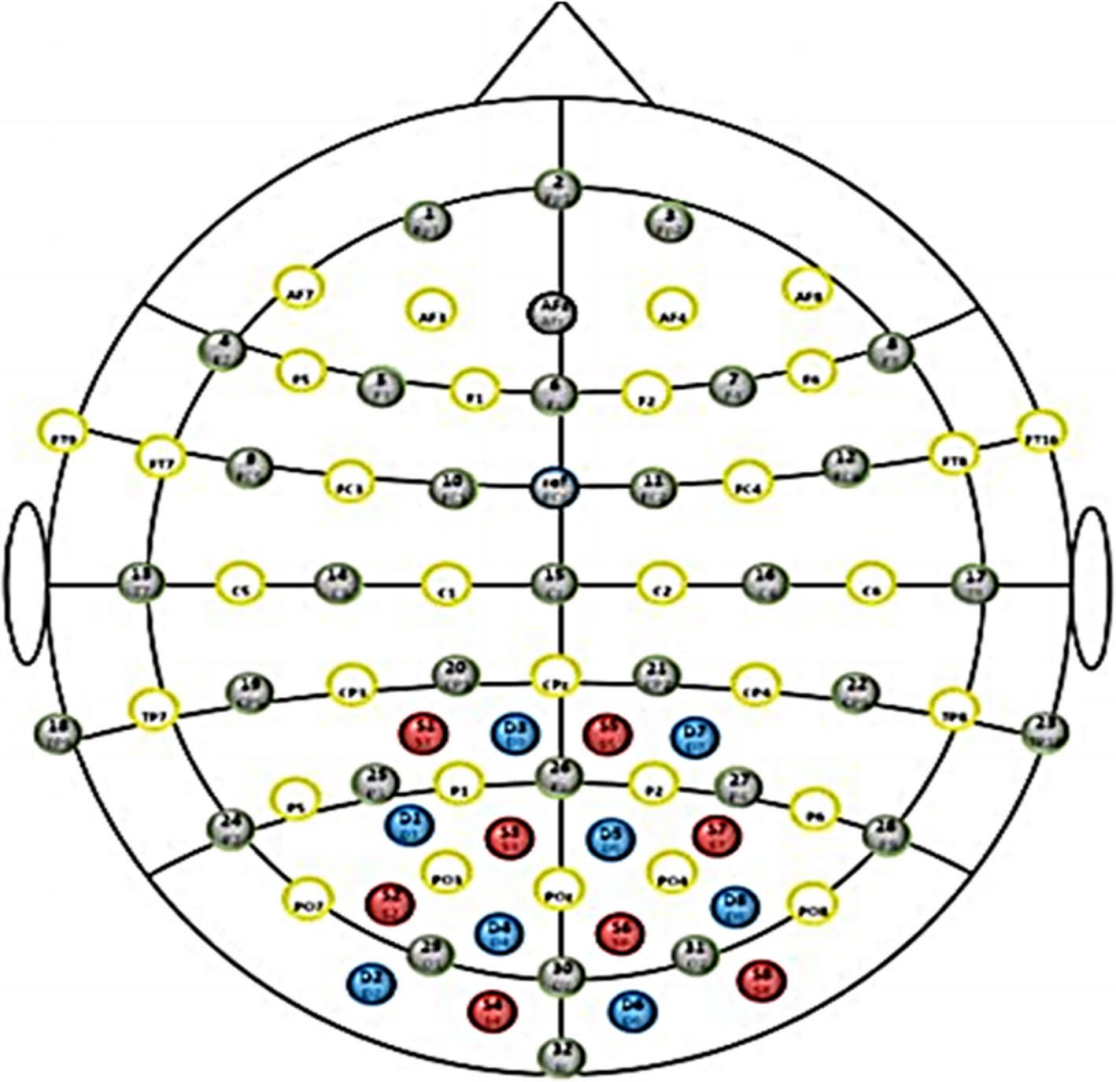
fNIRS monitoring with 16 channels in parieto-occipital area. The red circles indicate sources. The blue circles represent detectors

We used a continuous wave NIRS system (NIRSport 8X8, Nirx Medical Technologies LLC, Berlin, Germany). The fNIRS data acquisition software was the NIRStar 14.2 (Version 14, Revision 2, Release Build, 2016-04-15 NIRx Medizintechnik GmbH, Berlin, Germany; www.nirx.net). The fNIRS instrument included LED sources and photosensitive detectors. Each source employs two LEDs that emit a near-infrared light at 760 nm and 850 nm. The resulting sampling rate of fNIRS signal was 7.81 Hz. The arrangement of sources and detectors resulted in a total of 16 fNIRS measurement channels in parieto-occipital area, 8 for each side of hemisphere (Fig. 1).

The inter-optode distance was fixed at 30 mm, which is optimal to measure the hemodynamic activity variations over the cerebral surface. Each recording was preceded by a calibration procedure to verify that a good fNIRS signal acquisition was guaranteed. During the calibration procedure the NIRSport instrument was used to determine the signal amplification that each source–detector combination should have to achieve what is considered an optimal range (0.4 to 4.0 V) for the modulated raw signal level.

### fNIRS processing

The fNIRS signal processing was performed using nirsLAB (version 2017.6) running on MATLAB (version R 2013 b). The quality of the signals was evaluated by checking that the gain factor (indicating how much photo-current is amplified) and the coefficient of variation (the ratio between 100 times the standard deviation and the mean of the signal) were respectively lower than 8 and 7.5, two thresholds chosen during the calibration phase. If any channel did not pass the quality control, we attempted to improve the signal quality by making sure that the hair would be kept away of the light path. The signal processing was performed by firstly removing discontinuities. Using the fNIRS artifact removal package of MNE (version 0.24.1), we automatically identified common types of artifacts in fNIRS data (spike and baseline shifts) and we removed them with the Remove Spike Artifacts GUI of nirsLAB [21, 22]. The raw data were filtered in the band-pass 0.008–0.2 Hz to remove low oscillations such as respiratory and cardiac frequencies from fNIRS signal. In a preliminary evaluation, we observed hemodynamic activities in the 0.12-0.13 range during visual stimulation, so we argued they were not attributable to blood pressure artifact [23, 24]. The processed signals were then converted to optical intensities using the W. B Gratzer method (Med. Res. Council Labs, Holly Hill, London and N. Kollias, Wellman Laboratories, Harvard Medical School, Boston, MA, USA) and the optical intensities in turn were converted to oxyhemoglobin and deoxyhemoglobin concentration changes using the modified Beer-Lambert law [25]. Before computing the hemoglobin concentration changes, we carried out a baseline correction that was defined as the first 20 s of the total time of 60 s of resting state recorded before the visual stimulation.

**Fig. 2:**
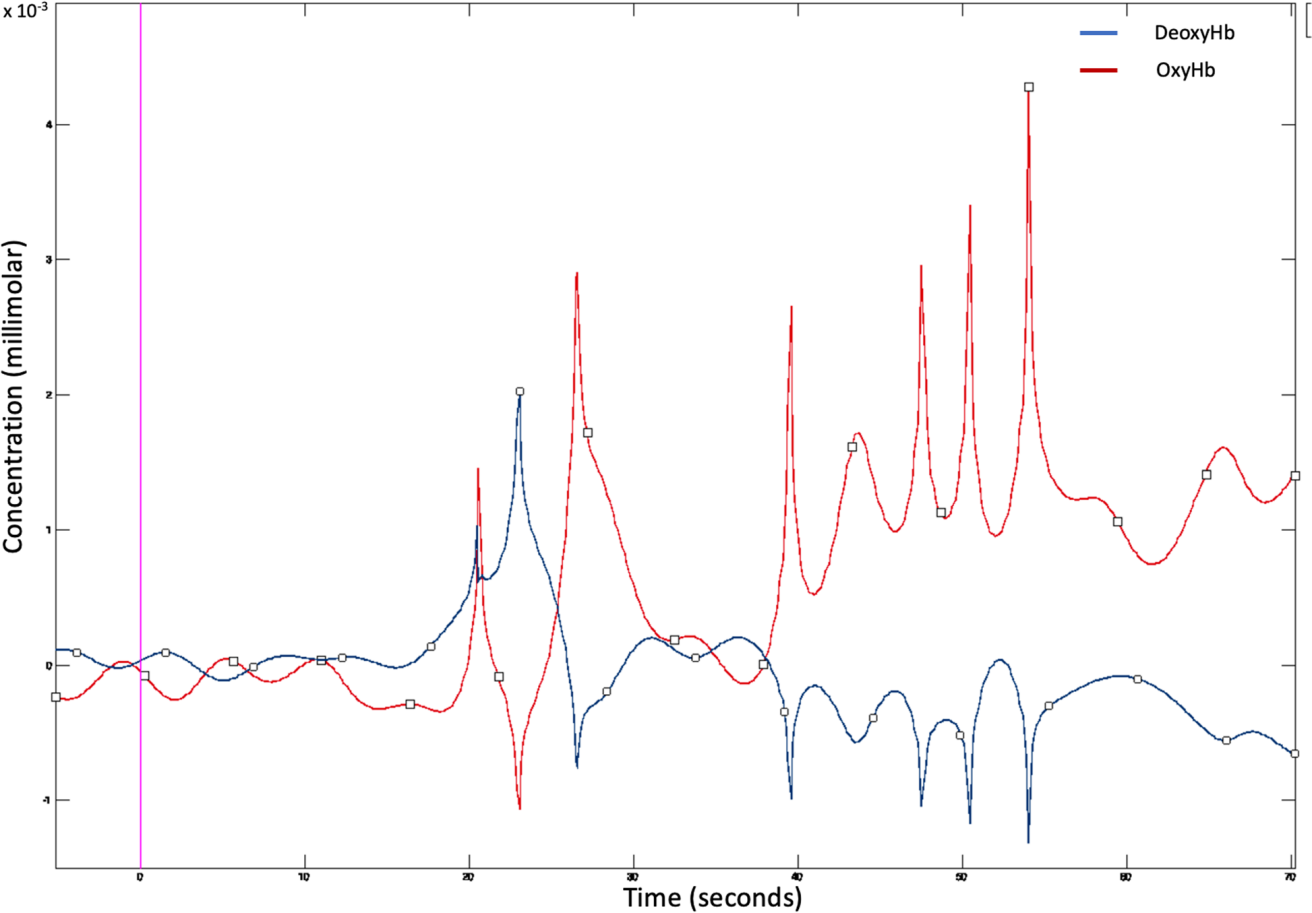
Deoxy (in blue) and oxyhemoglobin (in red) concentrations as a function of the time for one migraine patient at T0

As an example, Fig. 2 shows the oxy and deoxy hemoglobin trend as a function of the time for one patient at T0.

### Experimental procedure

**Fig. 3:**
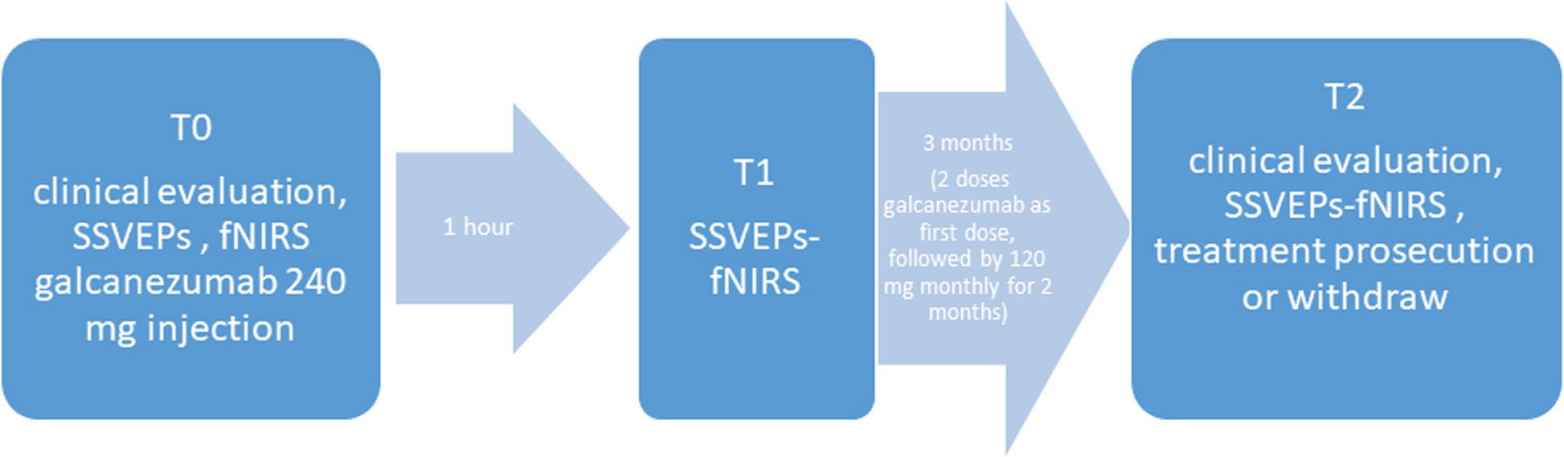
Experimental procedure

Giving that EEG/fNIRS co-recording under visual stimulation were never applied in migraine, the study size was not computable. SSVEPs and fNIRS were recorded before, 1 h and 3 months after the first dose of galcanezumab (240 mg), followed by a dosage of 120 mg monthly for 2 months (Fig. 3).

The study was conceived within the routine clinical practice, so patients underwent the EEG/FNIRS follow-up during the 3 month visit. They were also advised that the action on CGRP receptors could happen soon after drug administration, so SSVEPs recorded 1 h after the injection served as control session. The local Ethics Committee of Bari Policlinico General Hospital approved the study of SSVEPs and fNIRS in migraine patients, and all patients signed an informed consent prior to the recordings.

### Statistical analysis

Clinical features of migraine at T0 and T2 were compared using the Student’s t test for paired data.

For SSVEPs, the F, 2F and 3F power (μV [2]) was averaged over O1, O2 and Oz channels, and introduced in the ANOVA analysis for repeated measures with the condition T0, T1, and T2 as main factor, followed by Bonferroni test, according to SPSS program, version 28. In order to detect possible differences between episodic and chronic migraine, we also introduced migraine diagnosis, episodic vs chronic, as factor.

We evaluated the comparison of the SSVEPs in migraine in the different conditions with normal values from 10 controls, using the one way ANOVA analysis with the values of spectral power in F,2F and 3F as variables. In fact, we aimed to establish if basal SSVEPs anomalies in migraine could be reverted within normal values by galcanezumab.

In order to evaluate if the neurophysiological changes were an effect of clinical improvement, we correlated the percent rate of change between T0 and T2 of spectral power and headache frequency with the Pearson correlation test.

For topographical analysis and generation of Statistical Probability Maps, we applied the Student’s t test in order to perform paired comparisons of F,2F and 3F powers between T0 vs T2, T0 vs T1 and T1 vs T2.

In order to keep a uniformity among Statistical Probability Maps we applied a Student’s t test for unpaired data to compare the same spectral components between normal controls and patients in T0 and T2 conditions.

### fNIRS statistical analysis

To compute the degree of hemodynamic activation of each channel compared with the baseline, we used the Generalized linear model (GLM) implemented in nirsLab that, for the statistical analyses, relies on the Statistical Parameter Mapping 12 (SPM 12) tool. The GLM was carried out by choosing the Hemodynamic Response Function (HRF) to model the response during the visual stimulation. Finally, the results obtained from the GLM were used to evaluate, using the Student’s t test, if there were fNIRS channels wherein oxyhemoglobin changed in a statistically significant way (*p*-value < 0.05 corrected for multiple comparison) for the comparisons: T0 vs T2, T1 vs T2. In addition, we compared the patients with migraine at the time T0 and T2 with the control group. Single channels analysis included a one-way ANOVA among T0 and T1 vs T2 conditions, with the rate of migraine frequency reduction as covariate.

## Results

### Clinical features

All except 3 patients had a mild (30-50%) or relevant (more than 50%) reduction of headache frequency and intensity and MIDAS score (Table 1). One patient, BA, worsened in all headache scores, including MIDAS, thus, according to the Italian Health Public rules, treatment was discontinued. Results of Student’s t test are reported in Table 1, and show a general improvement of headache frequency, intensity and MIDAS, with reduced use of symptomatic drugs (Table 1).

At T0, recordings were performed 26.1 ± 3.2 h before and 27.2 ± 4.5 h after acute migraine. In one patient (DE) we stopped the recording session at T0 for the occurrence of migraine attack. Patient agreed to return 2 days after. At T2, patients underwent recordings 28.3 + 6.7 h after and 27 + 6.7 h before the next attack.

**Fig. 4:**
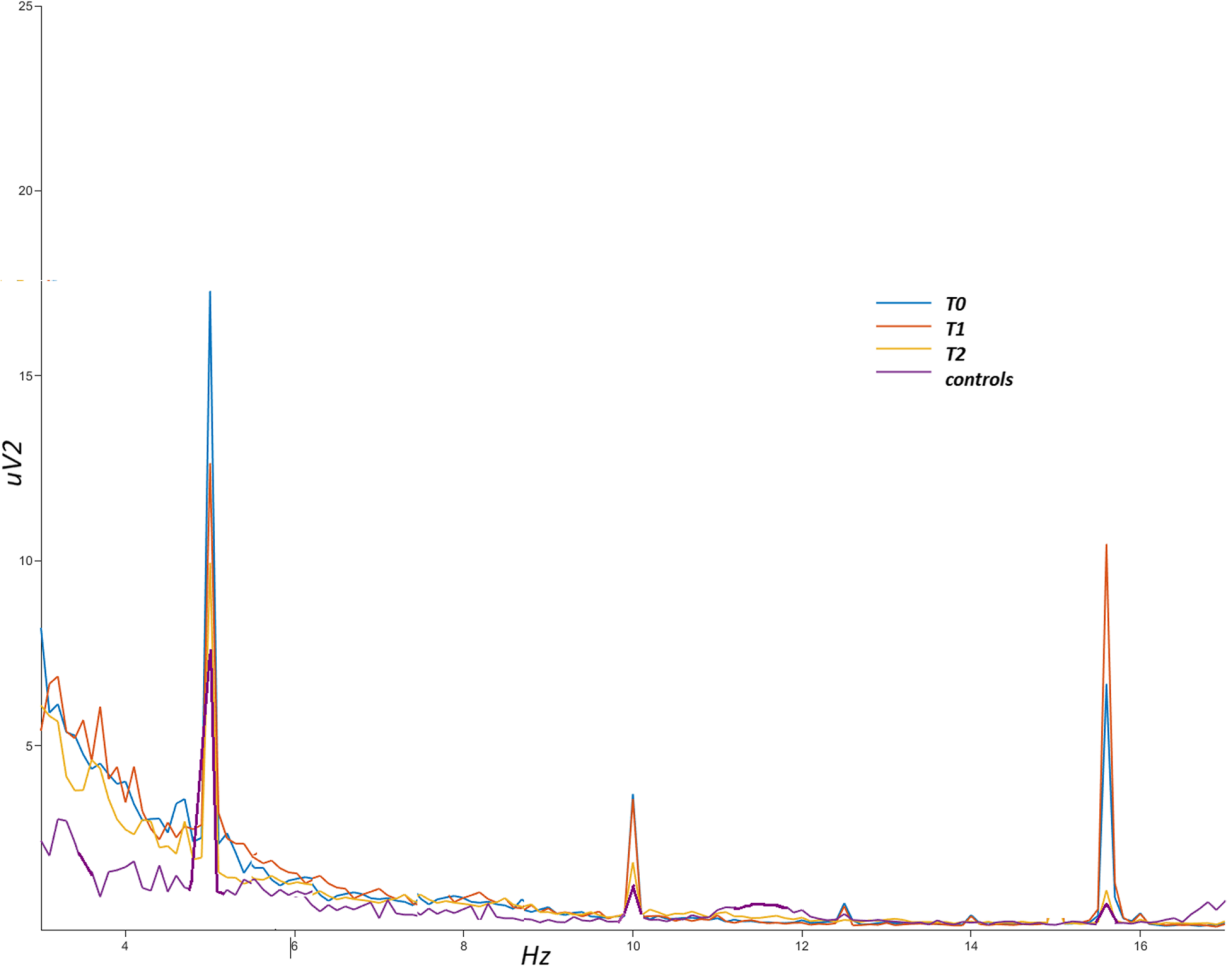
Grand Average of Spectral components F (5 Hz), 2F (10 Hz) and 3F (15 Hz) over the channels Oz, O1 and O2 in controls and migraine patients at T0, T1, and T2

**Fig. 5:**
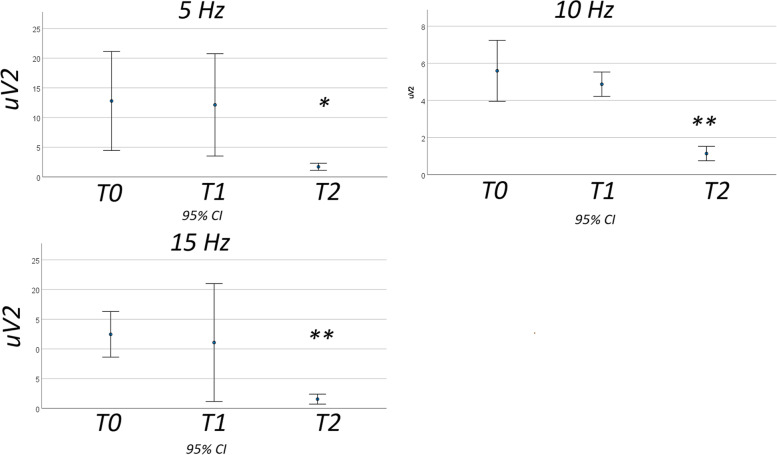
Mean and 95% CI of spectral power of F (5 Hz),2F (10 Hz) and 3F (15 Hz) elicited with 5 Hz stimulation in 13 migraine patients in basal condition (T0), after 1 h galcanezumab (T1) and 3 months galcanezumab (T2). Results of Bonferroni test are shown: T2 vs T0 and T1 * *p <* 0.05; ** *p <* 0.01; ++ T2 vs T0 *p <* 0.01

*SSVEPs* –single channels analysis- The ANOVA for repeated measured, aiming to detect changes within the migraine group for effect of treatment, showed a reduction of spectral power at T2, as compared to T0 and T1 conditions. At 5 Hz frequency, the F value (Roy square) was 5.99 (error degree 11, hypothesis degree 2), with a *p* value of 0.017. The Bonferroni test showed that at T2, the spectral power was significantly smaller than T0 and T1. At 10 Hz frequency, the F value was 71.82 (*p* < 0.001), with a clear reduction of spectral power at T2. At 15 Hz, we observed a reduction of spectral power in T2 condition (F 16.64, *p <* 0.001). The Bonferroni test was significant in the comparison between T0 and T2, and we obtained an approaching statistical significance between T1 and T2 (Fig. 4; Fig. 5).

The comparison of episodic migraine (7 cases) vs chronic migraine (6 cases) did not show significant differences (5 Hz migraine diagnosis DF 1: F 2.21 p 0.14, migraine diagnosis x conditions DF 2 F 0.66 p 0.52; 10 Hz: F 2.21 p 0.14, F 1.2 p 0.29; 15 H: F 1.8 p 0.17, F 1.3 p 1.22).

Considering the rate of headache days reduction at T2, we did not observe significant correlation with the rate of spectral power change in F, 2F and 3F frequency ranges (Pearson correlation: F-5 Hz − 0.117; 2F-10 Hz 0.38; 3F-15 Hz − 0.43, n.s.)

In the ANOVA model with F,2F and 3F as variables and the migraine groups at T0, T1,T2 and healthy controls as factors, we observed that at F-5 Hz frequency, spectral power values were different among groups (ANOVA F value 4.87 DF 3 p 0.005). The Bonferroni test showed a statistic difference in spectral power in the comparison between controls and migraine in T0 conditions (*p* < 0.05), and we obtained an approaching statistical significance in the comparison between controls and patients in T1 condition (p 0.071). At 10 Hz, the ANOVA F value was 34.84 (*p <* 0.001), and Bonferroni test was significant in the comparison between controls and migraine in T0 and T1 conditions (*p <* 0.05). At 15 Hz the ANOVA F value was 6 (*p <* 0.001), and the Bonferroni test showed that the spectral power was significantly lower in controls compared with migraine patients at T0 and T1 (*p <* 0.05).

### Topographic analysis

**Fig. 6:**
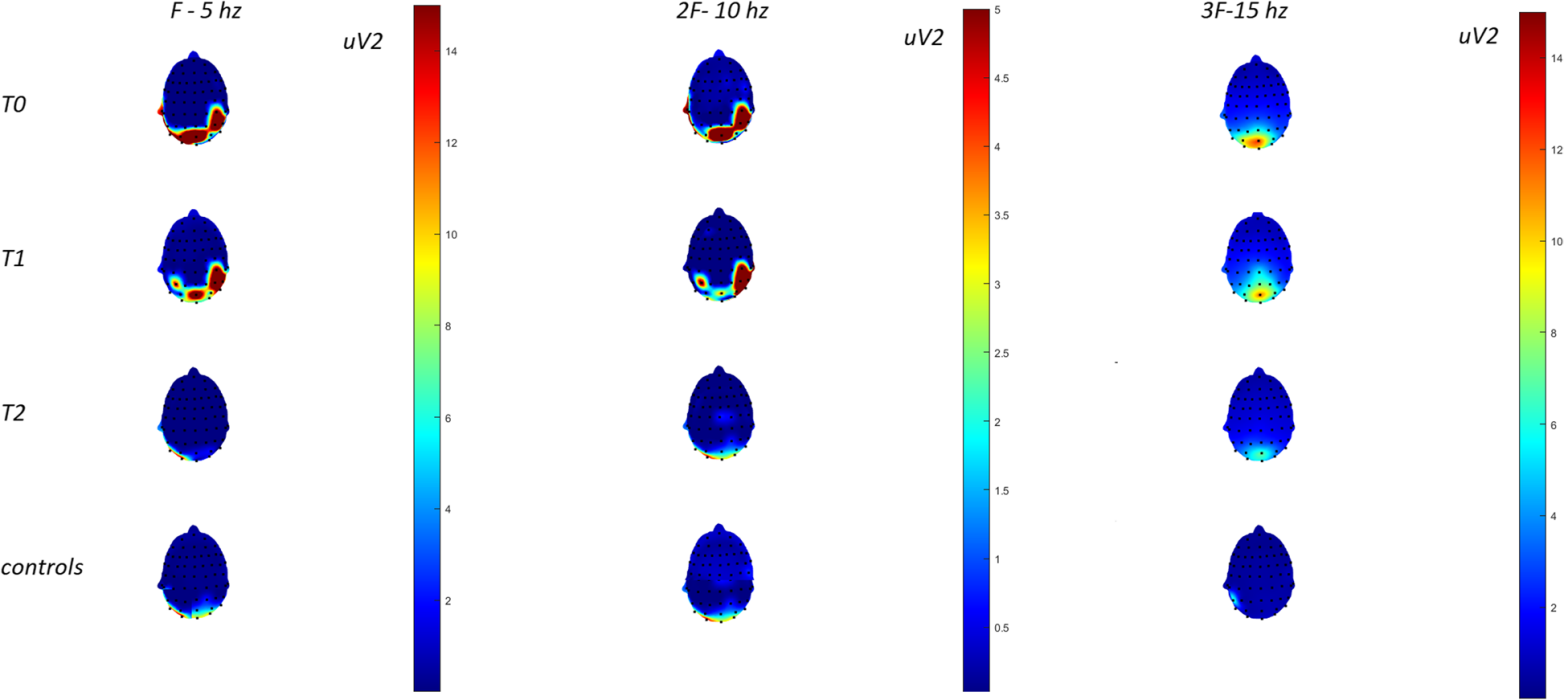
Topographic representation of SSVEPs in F (5 Hz), 2F (10 Hz) and 3F (15 Hz) in migraine patients (13 cases) at T0,T1 and T2 and controls (10 cases). The color map scale, emphasizes the prominence of spectral power in migraine patients at T0

We found a reduced cortical representation of F,2F and 3F spectral power in migraine patients after 3 months of GCA treatment. In Fig. 5, the parieto-occipital representation of SSVEPs at T0 was mildly attenuated after 1-h galcanezumab and clearly reduced after 3 months therapy. Healthy controls displayed a low amplitude occipital response at the evaluated frequencies, at least using the same color scale as applied in migraine patients (Fig. 6).

**Fig. 7:**
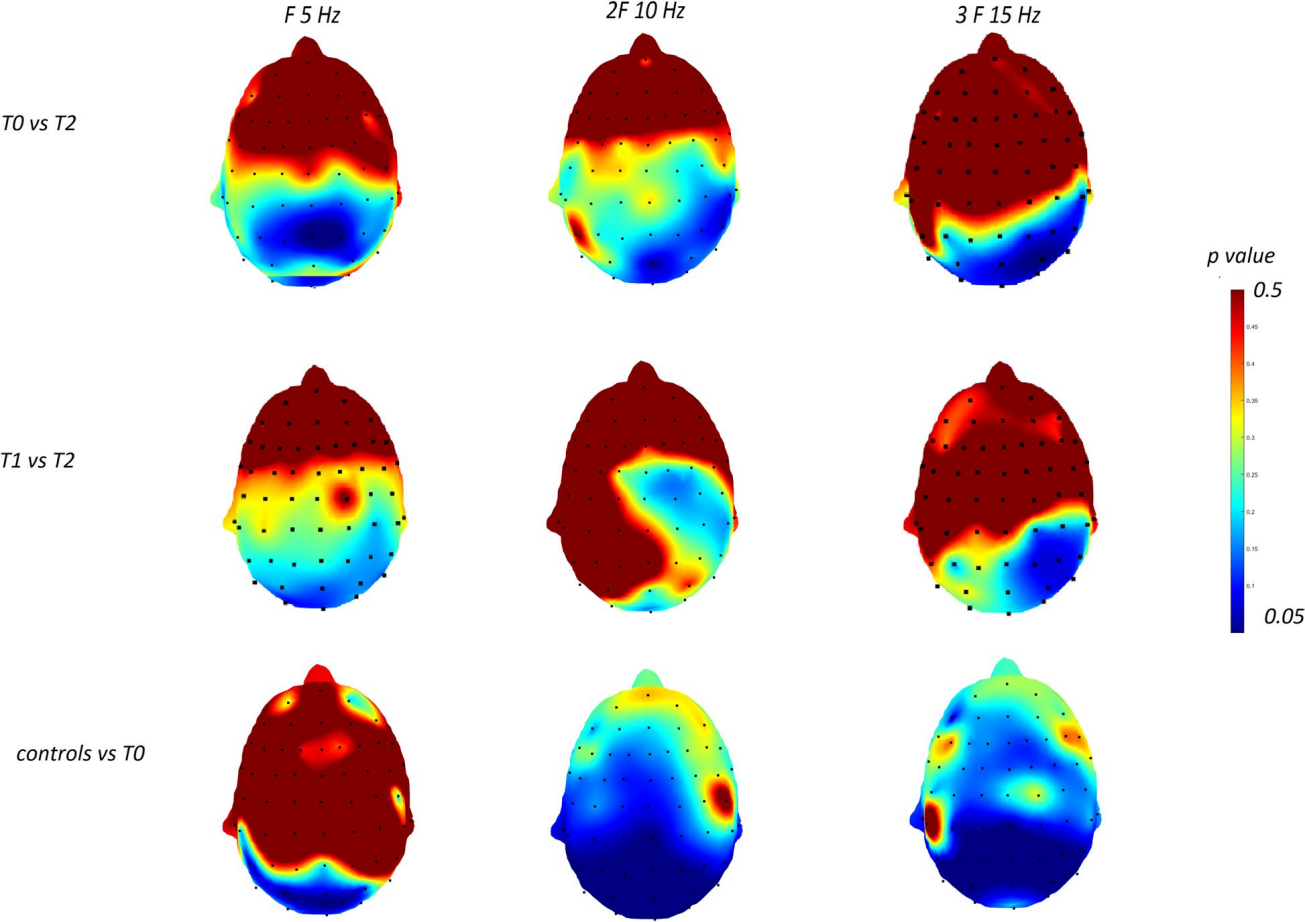
Statistical probability maps showing results of Student’s t test in the migraine group (13 cases) for the comparisons T0 vs T2, T1 vs T2, and migraine patients at T0 vs controls (10 cases). Blue colors express *p*-values lower than 0.05. The comparison between migraine patients in T2 and controls, was not significant

The t test showed that at T2, patients had a significant reduction of F,2F and 3F spectral power in respect to T0 and T1. The comparison with controls, was significant at T0 on parieto-occipital sites (Fig. 7).

### fNIRS analysis

**Fig. 8:**
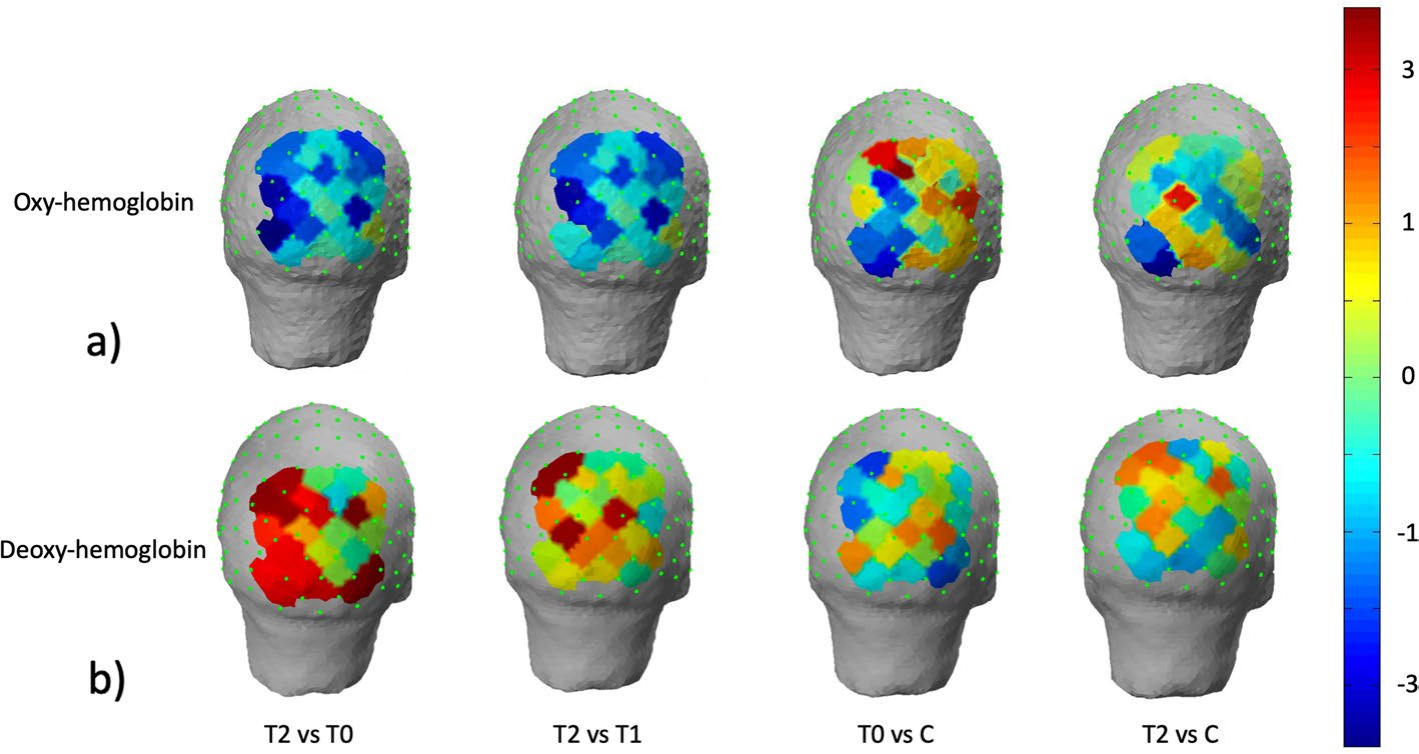
T-statistic map of the brain regions wherein there are significant differences in oxyhemoglobin a) and deoxyhemoglobin b) concentrations for the comparisons: migraine patients at T2 vs migraine patients at T0, migraine patients at T2 vs migraine patients at T1, migraine patients at T0 and at T2 vs controls. Intense blue color expresses significant reduction evaluated with paired Student’s t test, red color significant increase in migraine patients in basal condition compared to controls with the unpaired Student’s t test. The scale refers to t values

The unpaired Student’s t test, showed that migraine groups at the time T0 displayed an increase of oxyhemoglobin levels on several channels in respect to controls, with statistical significance on left parieto-occipital cortex (Fig. 8 a).

For the comparison with controls, we did not find any significant difference in oxyhemoglobin and in the deoxyhemoglobin levels at T2 (Fig. 8 a; Fig. 8 b).

**Fig. 9:**
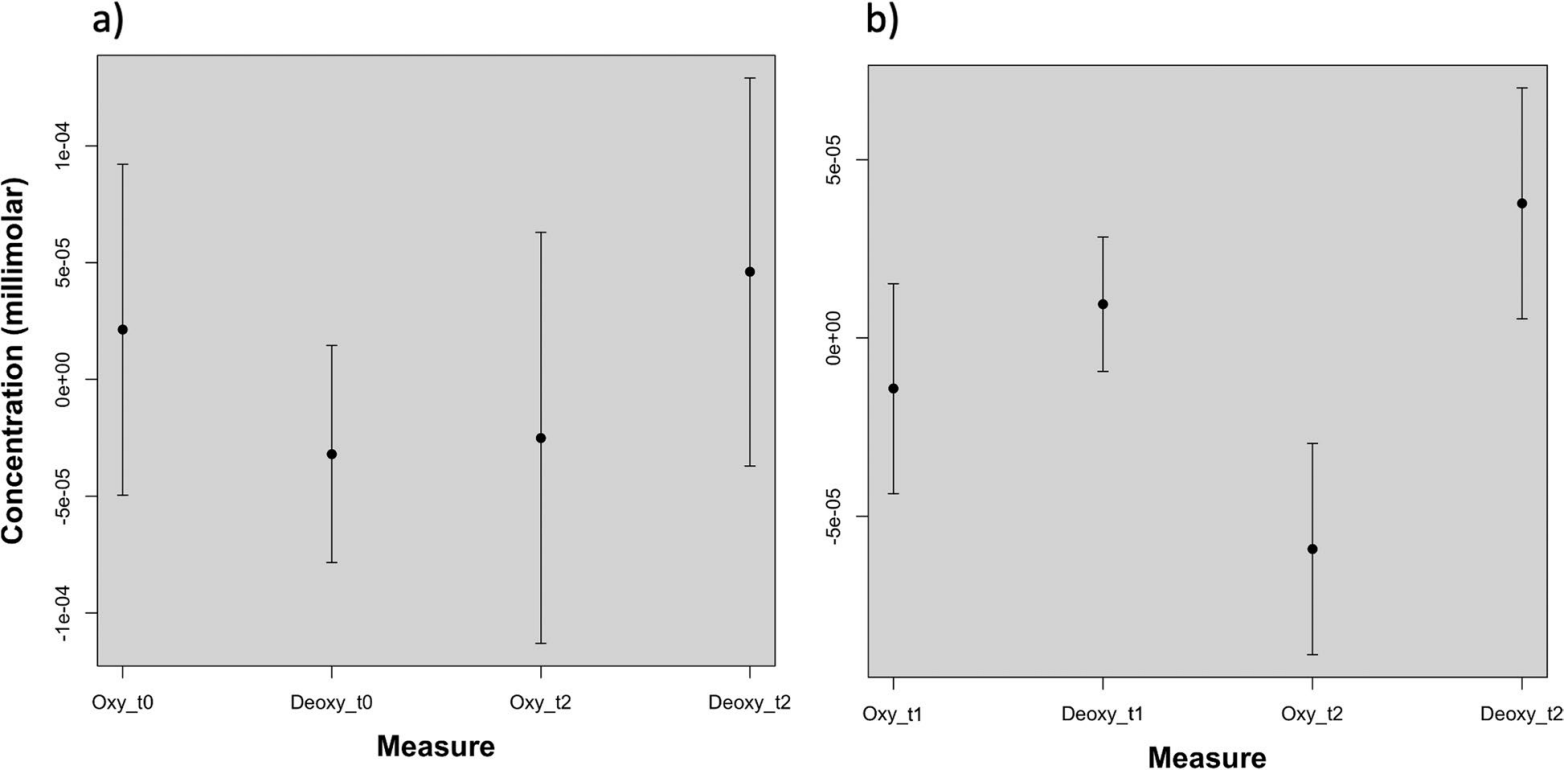
Plots reporting the values of oxyhemoglobin and deoxyhemoglobin levels averaged over the channels proved to be significant with the Student’s t test. In a) the comparison between T0 and T2 in b) the comparison between T1 and T2 (*p <* 0.01 for oxy and deoxyhemoglobin)

The paired Student’s t test showed a general reduction of the oxyhemoglobin concentration at T2, with significance on left occipital channels in respect to T0 and T1 and a general increase in deoxyhemoglobin at T2, with significance on left and right, and left occipital channels at T0 and T1, respectively (Fig. 8 a; Fig. 8 b, Fig. 9 a, Fig. 9 b).

The one way ANOVA analysis taking into consideration the average values of oxy and deoxyhemoglobin on the significant channels as variables, condition T0 vs T1 and T1 vs T2 as factors, and percent rate of headache reduction as covariate, showed no significant results (for oxyhemoglobin: T0 vs T2 F 7.219 p 0.013, covariate F 1.040, p 0.310; T1 vs T2 F 5.978 p 0.033; covariate: F 1.051 p 0.426; for deoxyhemoglobin: T0 vs T2 F 6.811 p 0.017; covariate F 0.232 p 0.636; T1 vs T2 F 8.775 p 0.008; covariate F 0.068 p 0.79757).

## Discussion

At the best of our knowledge, this is the first study attempting to evaluate the effects of Galcanezumab on the occipital cortex which is a crucial site for migraine pathogenesis. In this aim, we used a reliable method of repetitive visual stimulation, which exerts changes in EEG frequency domain and hemodynamic activity as well. We observed modifications in EEG spectral frequencies and oxy-hemoglobin levels as a result of the 5 Hz stimulation. These results suggest a medium term effect of GCA on cortical regions activated by visual stimulation, and could open a new scenario on the central effects induced by the peripheral modulation of CGRP transmission.

### SSVEPs findings

The increase of SSVEPs amplitude at specific frequencies, is a basal neurophysiological abnormality in migraine. Its recovery after 3 months treatment, could imply a direct action of galcanezumab on central mechanisms of migraine. The link between the occipital cortex abnormal rhythms reactivity and CSD and/or other factors producing migraine attack generation is still obscure. The thalamic-cortical dysrhythmia responsible for the different responses to repetitive visual stimulations, might be a predisposing factor to acute migraine [26].

The restoration of basal neurophysiological and hemodynamic abnormalities does not imply the clinical effect. In confirmation of the huge amount of clinical data about the beneficial effects of CGRP-mABs in resistant migraine, and GCA in particular [3], our patients showed on average a reduction in headache days, acute drugs consumption and disability. However, we observed 3 patients with a slight improvement, and even a single patient with migraine worsening. Moreover, the correlation between clinical and neurophysiological effects of galcanezumab, did not show relevant results. Our sample included both patients with chronic migraine and patients with episodic migraine. We did not observe differences in SSVEPs features between chronic and episodic migraine, neither in basal condition nor at the T2 follow-up, as both groups encompassed patients with long history of headache, drug resistance and high migraine frequency (more than 8 days/month) with possible pathophysiological similarities.

In previous studies, CNS acting drugs with proven efficacy on migraine, as topiramate, did not restore the abnormal occipital response to visual stimulations, differently from levetiracetam [27]. The correspondence between the specific action of drugs on neurophysiological features and clinical outcome is worthy of perspective studies in large groups. After 1 h of GCA injection, we observed a slight and not relevant reduction of SSVEPs. In fact, at 15 Hz, the difference of spectral power between T1 and T2 approached without completely reaching the statistical significance. Habituation phenomenon could have reduced the visual response after 1 h from the first repetition. However, we know that dis-habituation unlike habituation affects multimodal sensory responses in migraine [26]. SSVEPs are also scarcely influenced by habituation [28]. The lack of statistical relevance of this result seems not to confirm a short term effect of CGA on central processing of visual stimuli [29]. Anyway, after 3 months of treatment the inhibition of occipital visual reactivity was much more evident, as also showed by the hemodynamic and EEG differences between the T1 and T2 phases.

### fNIRS abnormalities in migraine

According to previous studies, in basal conditions, migraine patients exhibited augmented oxyhemoglobin levels in response to visual stimulations in comparison with controls [16]. This was the hemodynamic counterpart of the increased EEG response. In fact, the augmented fNIRS response to visual stimulations in migraine corresponded to the electrophysiological hyper-response of occipital cortex [16]. The CGRP-mABs have a direct vascular effect, reducing vasodilation in the periphery, e.g. in the meningeal districts, without inducing central vasoconstriction and changing cortical hemodynamic [30]. In our study, the hemodynamic parameters reverted into normal ranges with GCA treatment, a further confirmation that there was an inhibitory effect on occipital cortex reactivity [31]. This was independent from the clinical efficacy of the drug, confirming that the observed electrophysiological and hemodynamic phenomena would be a direct effect of the drug, rather than the indirect effect of migraine improvement.

### Why a peripheral anti-CGRP agent could affect occipital cortex reactivity?

The evidence that the used CGRP antagonist does not cross the blood barrier is well established [32], though CGRP receptors are located in many cortical and cerebellum areas [33].

The action of another CGRP antagonists –fremanezumab-was found ineffective on CSD generation, and partially efficacious on CSD propagation velocity [34]. Authors suggested caution in claiming a direct central nervous system effects of CGRP-mABs. There are no elements to suppose a different action mechanism for galcanezumab compared with other CGRP-mABs, as its molecular weight is not dissimilar from the others and could exclude blood brain barrier penetration [34]. On the other side, the lack of central passage of GCA across the blood brain barrier has been questioned [35].

The CGRP-mABs exert a potent antinociceptive effect, inhibiting trigeminal a-delta fibers and the vasodilation responsible for sterile inflammation [36]. We have recently demonstrated that cortical responses produced by the stimulation of a –delta afferents in the facial skin, are partially inhibited after short term effect of erenumab [5]. These results suggested a wide anti-nociceptive effect, including also a-delta afferents which are outside the trigemino-vascular system, as those located in the skin, involved in central sensitization phenomena as cutaneous allodynia. The modulation of cortical areas generating LEPs, opercular – insular cortex and anterior cingulate [37], could have a crucial role in restoring pain processing dysfunction and migraine chronification, but it does not explain the effect on migraine frequency reduction. Studies confirming Galcanezumab efficacy, included both migraine with and without aura patients [36], whose the first group surely affected by symptoms attributable to occipital cortex dysfunction and CSD generation.

However, these studies did not detail the specific effect on aura symptoms, and a possible clinical effect on symptoms attributable to occipital cortex involvement. The sole hypothesis we can postulate to explain our results, is that a wide and prolonged modulation of cortical areas involved in nociceptive signals elaboration, could exert a general influence on occipital cortex functioning and oscillation.

Russo et al. [38] observed that in migraine with aura, trigeminal noxious stimulation increased the bold signal of occipital cortex. It is plausible that the trigeminal inhibition due to Galcanezumab, could thus interfere with occipital cortex functions, even in migraine patients without aura.

Sensory integration between nociceptive system and visual responsiveness has been also recently postulated in patients with visceral pain [39], so the inclusion of occipital cortex within the network related to pain processing is increasingly in evidence. As a matter of fact, an indirect action of CGRP monoclonal antibodies on cortical regions located outside the pain network is the major hypothesis to explain [6]. In accord with this hypothesis, CGRP-mABs prevent, if efficient, not just the headache but also symptoms due to central hyper-reactivity such as photophobia, phonophobia, and prodromes, which likely makes a (at least partly) central effect, though mediated by peripheral action on nociception [35].

Recordings at T2, were done at the end of the 3rd month therapy, so the effect was cumulative of the entire therapeutic cycle and not related to the last injection. In this long-term modulation, a Blood Brain Barrier (BBB) passage of small mABC quantity cannot be completely excluded [35].

### Study limitations

We included few patients, though we verified the correspondence between clinical and neurophysiological effect.

A variability of spectral representation was present in normal controls, especially on the extra-occipital derivations.

Patients did not receive a real placebo treatment lasting 3 months. The ethical opportunity of a placebo controlled study aiming to show a neurophysiological phenomenon was questioned and the study design was realized within the routine clinical practice, without delay in drug prescription. An EEG/FNIRS follow-up 1 month after the first dose would be also interesting in light of a better definition of GCA mode of action and short-term effects.

The fNIRS limitations are numerous, especially the scarce intra-subject reproducibility and variability of the time course of hemoglobin levels under multimodal stimulations. The concurrent EEG analysis could reinforce the value of hemodynamic activity in the occipital cortex [40].

## Conclusions

The present study could contribute to add elements about a central effect of CGA, and CGRP mABs in general, in migraine patients. The increase of SSVEPs is a basal neurophysiological feature in migraine patients. The normalization of this neurophysiological feature indicates an action on the abnormal oscillations of the occipital cortex, and on the consequent cortical hemodynamic.

The observed phenomenon indicates that the potent and specific antinociceptive mechanism of GCA, other than modulating the cortical areas in the pain network, reaches other cortical targets with an important role in migraine pathogenesis.

If this is due to a passage of small parts of GCA across the BBB, or the counterpart of the constant inhibitory modulation of headache pain is not presently clear. The possible central effect of GCA could be slower than the peripheral mechanisms, and could explain the lack of correspondence with clinical efficacy after 3 months.

The general hypothesis that the peripheral modulation of brain structures devoted to elaboration of nociceptive afferents could reset abnormal cortical oscillators and consequent hemodynamic changes outside the main targets of inhibition, is attractive and testable in perspective studies on long term therapeutic effect.

## Data Availability

The datasets used and/or analysed during the current study are available from the corresponding author on reasonable request.

## Abbreviations

BBB: Blood Brain Barrier
CGRP: Calcitonin Gene Related Peptide
EEG: Electroencephalogram
fMRI: Functional magnetic resonance imaging
f-NIRS: Functional Near Infrared Spectroscopy
CGA: Galcanezumab
mABs: Monoclonal Antibodies
MOH: Medication Overuse Headache
SSVEPs: Steady state Visual Evoked Potentials
